# Unravelling the Homicide Drop: Disaggregating a 25-Year Homicide Trend in the Netherlands

**DOI:** 10.1007/s10610-021-09489-0

**Published:** 2021-06-18

**Authors:** Pauline G.M. Aarten, Marieke C.A. Liem

**Affiliations:** Institute of Security and Global Affairs, Leiden University, The Hague, The Netherlands

**Keywords:** Homicide, Decline, Trend, Homicide subtypes, Demographics, The Netherlands

## Abstract

The Western homicide drop is a known fact, but the reasons behind the drop have so far mainly focused on macro explanations. In this study, we argue that to understand the homicide drop, it is necessary to first explore whether the drop is general or specific. We do this by examining the subtypes of homicide together with perpetrator and victim demographic characteristics. This study seeks to describe the nature and scope of homicidal violence in the period 1992–2016 in the Netherlands, disaggregating by subtype of homicide, and perpetrator and victim gender constellation and age. In doing so, we make use of the Dutch Homicide Monitor. Findings show that the Dutch homicide drop is significantly related to homicides resulting from disputes and robberies and intimate partner homicides. The gender constellation and age distribution in all homicide types are further explored. This study highlights the importance of disaggregating data by subtype in unravelling the homicide drop.

## Introduction

For some time now, the homicide drop in the global West has become a well-established ‘fact’ in criminology (Aebi & Linde, 2014; LaFree et al., 2015; Weiss et al., 2016). Since the 1990s, the average homicide rate in Europe decreased by 37% (Lappi-Seppälä & Lethi, 2014). Even though the recent homicide decline has also been observed in North America and Australia (Weiss et al., 2016), it is important to note that this decline is not a universal phenomenon. Exceptions include countries in Central and South America that have shown a rise in homicide trends in the past decades (LaFree et al., 2015; UNODC, 2019). Other cross-comparative studies have confirmed that the decline is primarily a Western phenomenon (LaFree et al., 2015; Tuttle et al., 2018).

The reasons behind the Western homicide drop have dominated the research field of homicide studies (see, for example, Baumer & Wolff, 2014; Eisner, 2003; Koeppel et al., 2015; McCall & Brauer, 2014; Nivette & Eisner, 2012). It has now become particularly relevant, considering recent indications of an uptick in homicide figures in Scandinavia, such as in Denmark (Thomsen et al., 2019) and Sweden (Jürgensen, 2019), but also in the USA (see the special issue dedicated to this theme by Rosenfeld, 2019) and the UK (Clark, 2019). So far, scholars have mostly sought to explain variations in homicide patterns by resorting to strain, disorganization and social deprivation theories (Lappi-Seppälä & Lethi, 2014). More specifically, researchers have assessed how changes in macro-level factors, such as poverty and lack of opportunities, deprivation and inequality, social disorganization, strain and/or changes in (sub)cultural values create circumstances that can lead to changes in (homicidal) violence (e.g. Liem et al., 2012; McCall & Brauer, 2014; McCall et al., 2008; Pridemore, 2002). In these prior studies, each of these explanations has been explored using overall homicide rates. However, homicide cannot be seen as a monolithic phenomenon. Instead it has many manifestations, each of which is caused by different underlying characteristics and precursors (Ioannou & Hammond, 2015). For example, factors associated with intimate partner homicide have shown to be different compared to factors associated with non-domestic homicides (see Caman et al., 2016). This also means that the above-mentioned macro-level factors may not suffice when zooming in on specific subtypes of homicide, that is when disaggregating homicide by type.

Recently, scholars have highlighted the importance of examining subtypes of homicide in trend research (Kivivuori et al., 2014), or as Ioannou and Hammond (2015) point out, it constitutes “the next stage in its evolutionary lifespan” because “by examining the differences between the different types of homicide event, it is possible to unravel the complexities and subtleties of the crime of homicide to a far greater extent” (p. 159). According to Skott (2019), a disaggregation of the homicide trend is necessary to avoid erroneous conclusions known as ‘Simpson’s paradox’ (see also Hox, 2002). Simpson’s paradox is a phenomenon in which trends appear in several groups of data, but when the groups are combined, these trends disappear or reverse. Applied to the field of homicide research, a decrease of the homicide trend on an aggregate level does not necessarily imply a decrease in each of the subtypes of homicide. In order to provide an adequate explanation for the homicide trend and thus strengthen our theoretical knowledge in the field of homicide research, we need to take the complexity and heterogeneity of homicide into account.

## Disaggregating the Homicide Trend

Empirical evidence from numerous countries underlines the importance of disaggregating the homicide trend. Blumstein and colleagues (Blumstein et.al., 2000), for example, showed that the decline in American homicide could be ascribed to a decrease in young men involved in drug-related crimes. Several years later, Lehti (2014) disaggregated the homicide trend in Finland in the period 1998–2012 and found hidden countertrends within the overall homicide drop. The Finnish homicide drop appeared to be mainly the result of a decrease in alcohol-related homicides committed by working-age men between the ages 15 and 49. Skott (2019) studied the homicide trends on a disaggregated level in Scotland for the period 2000–2015. She found that the decrease in homicide in Scotland was primarily driven by public disputes involving young men using sharp weapons. However, she also concluded that domestic homicides demonstrated a relative increase over time, which could be attributed to a greater absolute reduction in the other subtypes of homicide.

Such studies that rely on disaggregated data—i.e. studying homicide according to subtype—also highlight the importance of taking gender and age into account when looking at homicide trends on a disaggregated level (see also Fox & Fridel, 2017; Fox & Piquero, 2003; Rennó Santos et al., 2019; Rosenfeld & Fox, 2019). The explanatory role of gender in the rise and fall of homicide rates is best explained using Veli Verkko’s (1967) static and dynamic laws. Taken together, Verkko argued that gender constellations play a significant role in homicide, as homicides largely involve male-to-male violence. Verkko’s static law states that most victims and perpetrators are male. Thus, when the prevalence of homicide is high, the proportion of female victims and perpetrators is low, and when the prevalence of homicide is low, the proportion of female victims and perpetrators is high. The dynamic law focuses on the change in time and assumes that when the homicide rate increases, this increase is mainly the result of an increase in male-to-male violence. Because, as Verkko explained, “The woman lives in a somewhat different and more peaceful atmosphere than the man and […] the factors influencing her, also, are not nearly as subject to changes as those affecting a man” (, 1951, p. 54). Even though Verkko formulated these laws based on his work on describing international homicide rates in the 1920s, recent homicide studies nonetheless still find support for Verkko’s laws (Gartner & Jung, 2014; Silverman & Kennedy, 1987; Trägardh et al., 2016), highlighting the need to examine gender constellations when studying homicide trends.

Although Verkko himself did not investigate the relationship with age, other researchers have argued that the reason behind the rise and fall in homicide rates can be primarily found in routines by young males (Aebi & Linde, 2014; Courtwright, 2001; Eisner, 2008). In their seminal paper, Aebi and Linde (2014) argue that elevated homicide levels in the second half of the twentieth century, compared to the first half, could be attributed to a combination of lifestyle factors that include later age of marriage, later parental age at the birth of their first child and the widespread availability of contraception. These factors, taken together, contributed to West European populations maintaining a single lifestyle until an older age. Such a lifestyle allows for more time to be spent in public places, thereby increasing the risk of both crime perpetration and victimization—including risk of homicide. Using the same logic described by the lifestyle theory, the authors suggested that the adapted lifestyles, particularly lifestyles of men between the ages of 30 to 40 years, made them more likely to spend their leisure time in supervised places such as shopping malls, cinemas and nightclubs (Aebi & Linde, 2014). The development and widespread availability of the Internet at the end of the 1990s and particularly in the early 2000s drastically increased the time spent indoors. Such a changed lifestyle amongst young men, in turn, has arguably lowered the risk of male-to-male homicide in public places, such as in cases of homicides that take place in the context of nightlife violence or robbery homicides. Thus, from this perspective, with a shift in lifestyles over time, we expect that the homicide drop is the result of a decrease in the risk of violent victimization and offending amongst young males in public spaces.

## Objectives

In line with recent contributions on the particularities surrounding country-specific homicide rates (see, for example, Lehti, 2014; Skott, 2019; Thomsen et al., 2019; Trägardh et al., 2016), we will first seek to describe the long-term homicide trend in the Netherlands and to examine how the overall trend relates to subtypes of homicide. Second, we explore the relationship between demographics—specifically gender constellations and age—and the trends in subtypes of homicide.

The Netherlands forms an excellent country to explore the homicide trend for two reasons. First, our analyses are based on vast and reliable data over a long period of time (1992–2016). Second, the Netherlands can be considered as representative for other Western European democracies, with similar homicide rates as its neighbouring countries (UNODC, 2019). Thus, in this exploration, we aim to add to our understanding of the mechanisms related to homicide trends.

## Research Context: the Netherlands

The Netherlands is a Western European country with a population of 17.3 million inhabitants (Statistics Netherlands, 2020a). Crime has been decreasing since the turn of the century. The highest rate was recorded in 2001–2002 with 93 crimes per 100,000 inhabitants, but since then, it has steadily decreased to 49 crimes per 100,000 inhabitants in 2016 (Statistics Netherlands, 2020b). In 2016, homicide constituted 0.3% of all registered crimes in the Netherlands (Statistics Netherlands, 2020b). The homicide rate peaked in the 1990s with a victimization rate of 1.83 per 100,000 inhabitants, but since 2003, this rate has decreased to 0.62 victim per 100,000 inhabitants in 2016 (Aarten et al., 2019). According to the UNODC (2019), the Netherlands has one of the lowest homicide rates in the world, similar to homicide rates in Switzerland and Norway.

In comparison with countries such as the UK and the USA, the Netherlands does not have a long history of homicide research (Kivivuori et al., 2014). Only in the past 15 years has there been an increase in scholarly attention for the most severe type of violence. This increase has resulted in the Netherlands being one of the largest contributors to the academic field of homicide in Europe (Kivivuori et al., 2014). However, little research has been done on long-term trends of lethal violence, let alone on a disaggregated level. So far, Dutch scholars have mainly focused on its epidemiology (e.g. Aarten et al., 2019; Liem & Leissner, 2016; Liem et al., 2012; Nieuwbeerta & Leistra, 2003; Smit et al., 2001), but also on subtypes of homicides and their perpetrators and victims (e.g. Aarten & Van der Laan, 2012; Liem, 2010; Liem & Haarhuis, 2015; Liem & Koenraadt, 2018; Van der Port, 2001), and punishment and recidivism (e.g. Baay et al., 2012; Van Wingerden & Nieuwbeerta, 2010; Vries et al., 2010).

To the best of our knowledge, only three previous studies have specifically assessed the Dutch homicide trend. The first study, by Nieuwbeerta and Leistra (2003), focusing on a 10-year period (1992–2001), found an overall downward trend in homicide starting at the end of the 1990s. They did not, however, disaggregate the homicide trend according to subtype but only looked at the prevalence of subtypes. In 2012, Liem and colleagues (Liem et al., 2012) examined the overall homicide trend in the period 1992–2009 and sought to explain the reasons for this recent drop by resorting to societal developments. Changes in homicide clearance and changes in unemployment were both significantly associated with the drop. They also noted that besides an overall drop, there was a decrease in all subtypes of homicide in the period 2004–2009, with the exception of the category ‘other or unknown homicides’. However, they did not further explore the reasons for the drop on a disaggregated level. A further investigation by Ganpat and Liem (2012) showed that all homicide subtypes in the period 1992–2009 showed a downward trend from 2003 onwards. In other words, results so far have shown no rise or fall of a specific type of homicide compared to other subtypes of homicide. They described several hypotheses that could explain these findings, including changes in the demographic composition of society, social disorganization and economic deprivation. Yet, these explanations were not formally tested.

To summarize, only two studies have described the Dutch homicide trend on a disaggregated level. Yet, since their (main) focus was on outlining the epidemiology of homicide in the Netherlands, the homicide trend on a disaggregated level only formed a subsection of each study. To partake in the next stage of the evolutionary lifespan of homicide research (Ioannou & Hammond, 2015) and learn more about the Dutch homicide trend, this study will be the first in the Netherlands to explore the homicide trend on a disaggregated level. Furthermore, it stands apart from the above-mentioned studies by being able to study the homicide rate on a disaggregated level over a 25-year period. And finally, it will delve into the homicide trends by looking specifically at the relationship between demographics and each homicide subtype.

## Methods

### Data

This contribution makes use of the Dutch Homicide Monitor (DHM), a monitoring system that captures detailed information on all homicides in the Netherlands in the period 1992–2016 (Aarten & Liem, 2021). Prior to 1992, there was no nationwide uniform registration of homicide cases. The DHM is part of the European Homicide Monitor (EHM), a European-wide data collection initiative, that follows a uniform structure (i.e. uses the same variables and values) (Granath et al., 2011). The DHM relies on a total of six sources, altogether seeking to provide a complete and valid overview of all homicides that occurred in the time period studied. These sources include (1) newspaper articles and auxiliary public domain sources, (2) annual homicide overviews by the magazine Elsevier, (3) information from the National Police, (4) information supplied by the public prosecutor’s office, (5) detailed information stemming from criminal justice records and, where available, (6) information retrieved from perpetrator’s forensic mental health reports (for more information, see Aarten & Liem, 2021).

### Inclusion Criteria

Using the same definition as the EHM, to allow for international comparisons, the DHM considers homicide as an intentional criminal act of violence, which results in the death of one or more individuals. This definition covers all murders, (involuntary) manslaughters and infanticides. Attempted homicides, suicides, abortion, euthanasia and assistance with suicide are not included in the data (see also Granath et al., 2011; Liem, et al., 2018b).

### Variables

#### Subtype

In order to examine the trends of subtypes of homicide, we followed the validated coding manual of the EHM (Granath et al., 2011) as well as prior international research in this area (Liem et al., 2018b; Suonpää et al., forthcoming; Thomsen et al., 2019). Here, homicide is categorized according to a combination of victim-perpetrator relationship and homicide motive (see also Granath et al., 2011). In case there was an overlap between subtypes—for example, an uncle killing his niece after sexually abusing her—the relationship between victim and perpetrator (domestic homicide) took precedence over the motive. If the relationship between victim and perpetrator was a non-domestic one, the motive constituted the primary source of classification, i.e. sexual homicide.

The EHM-variable type of homicide includes 15 values. These values were reduced to seven (more general) subtypes in this study, while still taking the victim-perpetrator relationship and motive into account: (1) intimate partner homicides; (2) other domestic homicides (including child homicide, infanticide and other familial killings); (3) homicides resulting from a non-criminal dispute (including nightlife violence)^1^; (4) criminal homicides that include homicides in the context of organized crime and drug trade; (5) robbery homicides that include street robberies and commercial and residential robberies; (6) other homicides, characterized by a profound influence of mental illness and sexual homicides; and (7) unknown homicides, including cases where both the relationship between victim and perpetrator and the motive remained unknown. This reduction in categories was necessary as some subtypes, such as sexual homicide and homicides characterized by a profound influence of a mental illness, amounted to too few cases to otherwise draw meaningful conclusions. However, unlike previous research colleauges (e.g. Liem et al., 2018b), in this study, we separated intimate partner homicide from other domestic homicides. Previous research has shown that intimate partner homicide is the most common type of domestic homicide (Stöckl et al., 2013) and has different risk factors than other types of (domestic) homicide (e.g. Caman et al., 2016). For this reason, we included intimate partner homicide and other domestic homicides as separate categories.

#### Gender Constellation

Further, we created a gender constellation variable that combined perpetrator gender with victim gender (male-to-male, male-to-female, female-to-male and female-to-female). In 962 cases that involved multiple perpetrators, the constellation was based on each victim and the principal perpetrator per case. The principal perpetrator is defined as the perpetrator who had been prosecuted for homicide. If more than one perpetrator was prosecuted for homicide, then the principal perpetrator was the one receiving the most severe sanction. If sanctions were equal, then the perpetrator with the closest relationship to the victim was identified as the principal perpetrator. If this information was not available, or if the perpetrators were equally close to the principal victim, then the principal perpetrator was chosen at random. The combination was not calculated for uncleared cases, i.e. cases for which there was no suspect known to the police, or cases where the gender of either the principal perpetrator or victim was unknown. These victims (in total 18.7% of the 5170 victims) were excluded from gender-specific analyses.

Of the total 4849 cases, 826 cases (or 17%) remained uncleared. The number of uncleared cases per year lies around 4%, but this percentage is higher in the earlier years (1990s) compared to the recent years (2010s). This is most likely the result because the earlier cases are old cases where investigation strategies were not as high-tech compared to the recent years. In these uncleared cases, 865 victims died. Based on information retrieved from media reports and police data, we were able to extract a few background characteristics of these cases. Of these victims, 82% were male, 17% were female and 1% of the victims’ gender remained unknown. Most victims were between 20 and 50 years, with a median age of 26 years. While more than half of the cases remained unclassified in terms of typology, based on the media and police information, we were able to identify nearly a quarter of these cases as criminal milieu cases. Around 8% of the cases were robbery homicides, and 6% were dispute homicides. The remaining cases were either other homicides (2.7%) or familial homicides (2%). We realize that basing our analysis on cleared homicides means that we are basing our conclusion on a selection of homicides, as homicides are not cleared at random. We will further discuss this limitation in our discussion and conclusion.

#### Age

When analysing the disaggregated data, we calculated the median age of the victims and principal perpetrators per year. We only looked at the age of the principal perpetrator, instead of the age of all perpetrators, as the analyses on gender constellation also only included principal perpetrators.

When calculating the age, the median is preferred, because it provides a better picture of the age distribution in homicide than the calculated average as it is not affected by outliers. The relatively small number of cases did not allow us to combine gender constellation with age per timeframe, and for this reason, these two demographic variables were analysed separately in this article.

### Analyses

To assess the nature and scope of homicide over a 25-year period, we first calculated the homicide victimization rate per year. In doing so, we relied on total population size per year (retrieved from Statistics Netherlands, 2020a). We also calculated the total gender constellation percentages per year. Furthermore, we calculated the homicide rate per age group for all perpetrators and victims (less than 20 years, 20–39 years, 40–64 years, 65–79 years and 80 years and older), hereby also taking the total population size of each age group per year into account (retrieved from Statistics Netherlands, 2020a).^2^ These analyses set the scene of the overall homicide trend in the Netherlands in the period 1992–2016.

In the remaining analyses, we focused on disaggregated homicide rates. The disaggregated homicide rates were also calculated by taking the population size per year into account. However, low annual homicide counts are more susceptible to outliers, and for this reason, 3-year moving averages of each annual rate were calculated. To determine whether the trend of each subtype was related to the overall homicide rate, Spearman’s correlations were calculated. Furthermore, we calculated the gender constellation percentages and calculated the median age of the victims and their principal perpetrator per subtype of homicide. Here too, we calculated Spearman’s correlations to determine whether the observed trend was statistically significant.

For this article, findings are displayed in graphs. To increase the transparency of these graphs, the 25 years was split into eight periods (1992–1994, 1995–1997, 1998–2000, 2001–2003, 2004–2006, 2007–2009, 2010–2012, 2013–2016). Annual trends and demographics were averaged for each period. These eight periods were chosen for two reasons. First, we followed Skott’s (2019) reason that a division into periods avoided a small *n* while maintaining as much variance as possible. Second, the study period could be easily divided into eight periods, allowing the same number of years in each period except for the final period (which comprised of 4 years). Spearman’s correlations were calculated between demographics and the years of observation.

## Results

### The Overall Homicide Trends

**Fig. 1:**
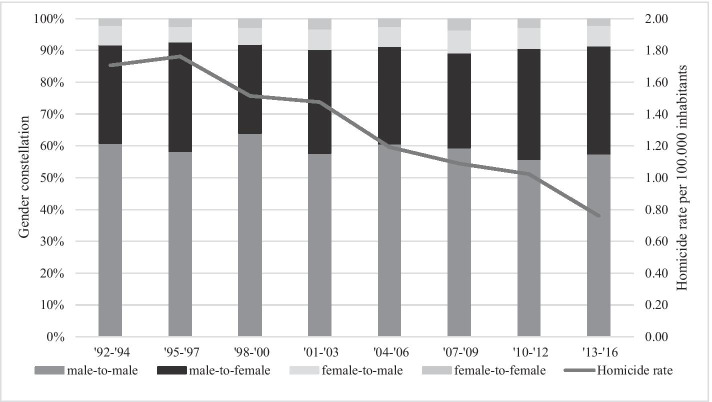
Overall homicide rate per 100,000 inhabitants and percentage gender constellation in the period 1992–2016

Figure 1 shows the overall homicide rate per 100,000 inhabitants and the gender constellation of cases in the period 1992–2016. In the 25-year time period, a total of 4849 homicide cases took place, resulting in 5170 lethalities. Throughout the study period, there has been a decline in homicide mortality, both in absolute numbers and in population-adjusted rates. High homicide mortality rates in the early 1990s, averaging at 1.71 victim per 100,000, increased slightly to 1.76 in the years 1995–1997 before declining for the remainder of the years and reaching its lowest rate of 0.76 victim per 100,000 inhabitants in the years 2013–2016.

The bars in Fig. 1 (and the corresponding Tables 1 and 2 in the Appendix) display the gender constellations of the principal perpetrator and their victims. The most common gender constellation throughout the study period was male-to-male homicide, which remained considerably stable over time and constituted, on average, 59% of all cleared homicides. The second largest group (male-to-female homicide) increased over time, averaging at a third of all cleared homicides in the final periods. The third largest gender constellation (female-to-male homicides), averaging at 6% of all cleared homicides, also shows a small increase over time. The smallest group consisted of female-to-female homicides (on average, 2.6% of all cleared homicides) and showed a stable trend over all eight periods.

**Fig. 2:**
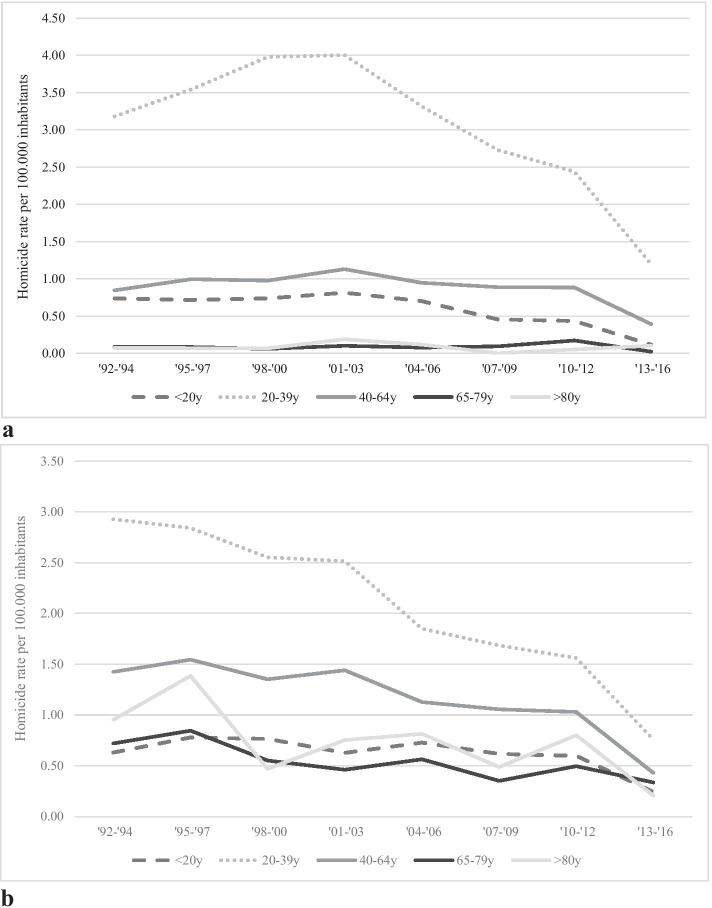
**a** Homicide rate of perpetrators per age group per 100,000 inhabitants in the period 1992–2016. **b** Homicide rate of victims per age group per 100,000 inhabitants in the period 1992–2016

Figure 2a and b displays the homicide rates per age group of the perpetrators and victims. Both figures show a decline in nearly all homicide rates in all age groups, suggesting that the homicide drop was not specifically related to a certain age group. However, as the general Dutch population has increased considerably in the past 25 years, with a 53% increase in the age group 80 years and older and only a 9% increase in the age group 20–39 years old, two findings are noteworthy. For both perpetrators and victims, the largest decline in homicide rate was in the age group 20–39 years. For perpetrators in this age group, the homicide rate was 3.18 per 100,000 inhabitants in 1992–1994 and declined to 1.20 per 100,000 inhabitants in 2013–2016. For victims, the rate was nearly 3 per 100,000 inhabitants in the 1990s and declined to 0.76 per 100,000 inhabitants in the final years of the observation period. The only exception are the perpetrators in the age category 80 years and older, where we see an increase in homicide rate. However, since this is a very small group (*n* = 13), we need to be careful in drawing conclusions.

### Disaggregating the Overall Homicide Trend

**Fig. 3:**
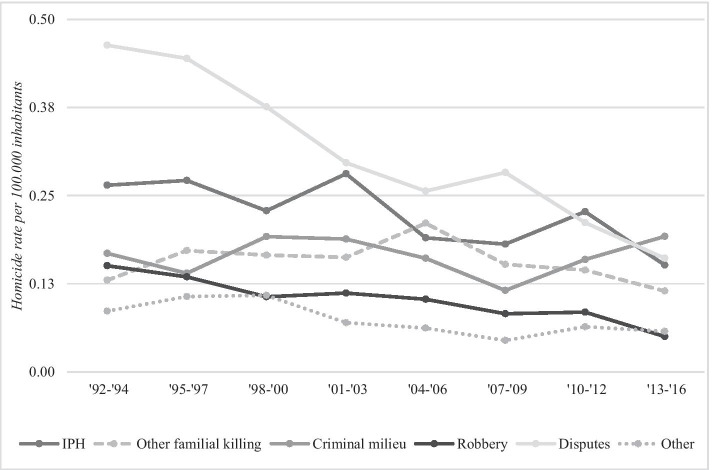
Disaggregation of homicide rate per 100,000 inhabitants by subtype, in the period 1992–2016

Figure 3 reflects the homicide trend disaggregated by subtype. While most homicide subtypes showed a decreasing trend over the eight periods, the overall homicide trend was most strongly associated with a decrease in dispute homicides (*r*_s_ = 0.93, *p* = 0.000), followed by robbery homicides (*r*_s_ = 0.81, *p* = 0.000) and intimate partner homicides (*r*_s_ = 0.72, *p* = 0.000). Dispute homicides showed the largest drop over the years compared to the other subtypes, declining from 0.46 victim per 100,000 inhabitants in the period 1992–1994 to 0.16 victim in the period 2013–2016. Robbery homicides decreased to a somewhat lesser extent: from 0.15 victim in the first period to 0.05 victim per 100,000 inhabitants in the final period. Intimate partner homicide also decreased in the 25 years but showed more fluctuation in their trends, with peaks in the periods 2001–2003 and 2010–2012 (see also Tables 3 and 4 in the Appendix).

While the category ‘other homicides’ was also significantly related to the overall homicide trend (*r*_s_ = 0.75, *p* = 0.004), this was mainly the result of a decrease in sexual homicides. Homicides primarily characterized by a profound influence of mental illness were not significantly related to the overall homicide trend (*p* = 0.166). However, this category was altogether too small to formulate meaningful conclusions and was therefore excluded in further analyses.

Other familial homicides and criminal milieu homicides were not significantly related to the overall homicide trend. Even though the rate of other familial homicides decreased slightly over time (from 0.13 victim per 100,000 inhabitants in 1992–1994 to 0.11 victim per 100,000 inhabitants in 2013–2016), the decrease was not as strong as the decrease in other subtypes and (only just) not significantly related to the overall drop (*r*_s_ = 0.37, *p* = 0.070). Criminal milieu homicides, in contrast, slightly increased over time (from 0.17 victim per 100,000 inhabitants in 1992–1994 to 0.19 victim per 100,000 inhabitants in 2013–2016), which could explain why the trend was not significantly related to the overall homicide trend (*r*_s_ = 0.16, *p* = 0.438).

### The Relationship Between Gender Constellation, Age and Homicide Subtypes

**Fig. 4:**
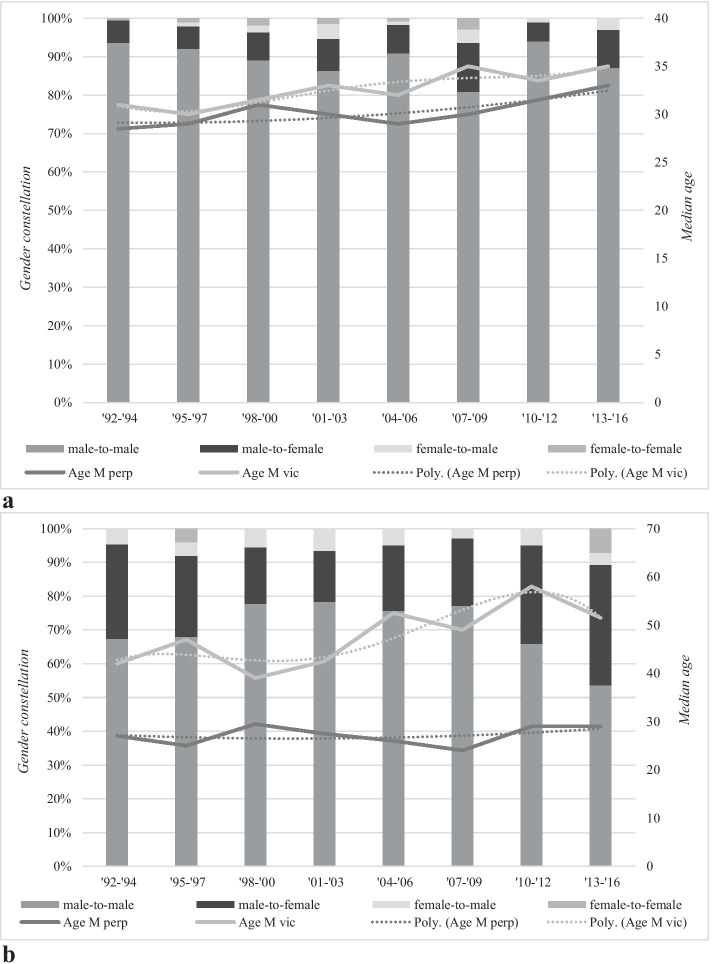

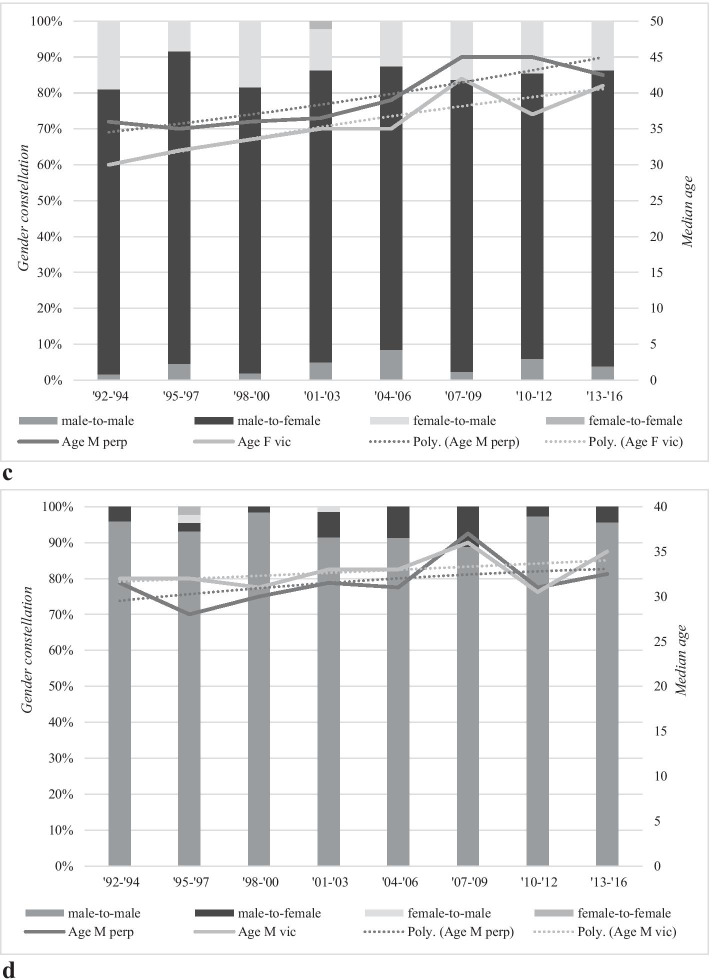

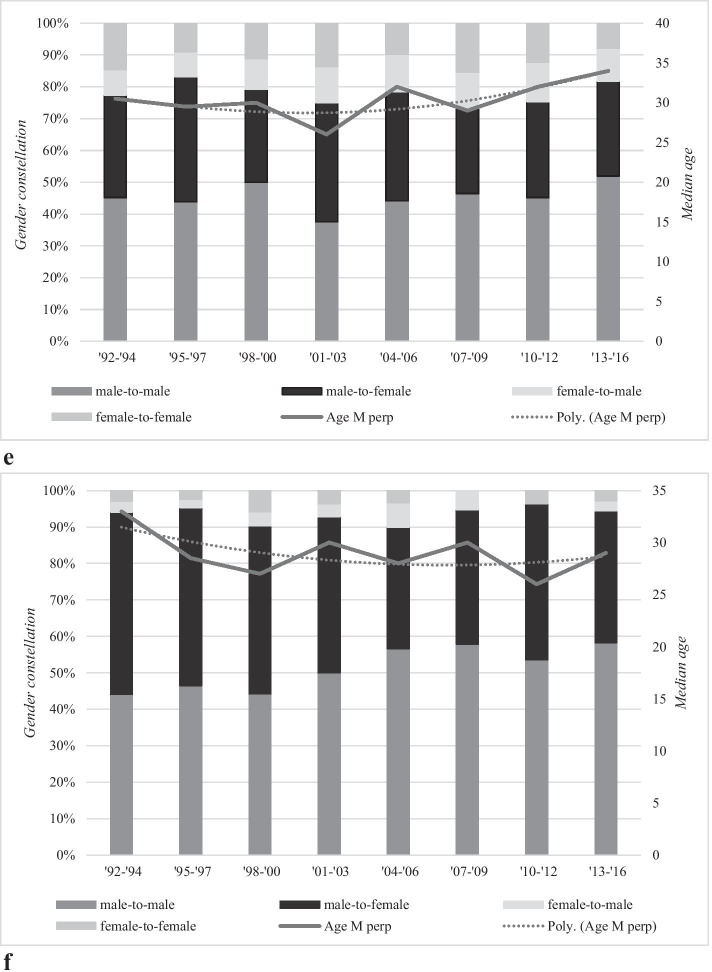
**a** Median age and gender constellation of dispute homicides in the period 1992–2016. **b** Median age and gender constellation of robbery homicides in the period 1992–2016. **c** Median age and gender constellation of intimate partner homicides in the period 1992–2016. **d** Median age and gender constellation of criminal milieu homicides in the period 1992–2016. **e** Median age and gender constellation of other familial homicides in the period 1992–2016. **f** Median age and gender constellation of other homicides in the period 1992–2016

In Fig. 4a–f, the relationship between gender, age and the six homicide subtypes is further explored. We will first discuss the three subtypes that were statistically significantly related to the overall homicide rate: dispute, robbery and intimate partner homicides.

Figure 4a (see also Table 5 in the Appendix) shows the relationship between gender and age with dispute homicides. Most disputes took place between males (on average, 89% in the 25-year period). Over time, the proportion of male-to-male homicides gradually decreased from 93.7% in the first years of analysis (1992–1994) to 80.9% in the period 2007–2009, before showing a strong uptick in 2010–2012 and finally averaging at 87.1% in the period 2013–2016. This decrease is, however, not statistically significant (*p* = 0.16). The trendline of age of both male perpetrators and male victims shows a slight increase throughout this time: At the start of the observation period, both male victims and male perpetrators were in their early 30s, and by the end of the 25 years, the median age of the male victims and male perpetrators increased to mid-30 s. This increase is statistically significant for male perpetrators (*r*_s_ = 0.52, *p* = 0.01), but not for male victims (*p* = 0.08). Approximately 8% of all dispute homicides took place between a male perpetrator and a female victim (see Fig. 4a). The absolute size of the male-to-female dispute homicides increased throughout the observation period, from 5.8% in the early 1990s to 12.8 in the period 2007–2011, before it declined to 10.1% in the period 2013–2016, but this increase is not statistically significant (*p* = 0.30). Dispute homicides between female perpetrators and male victims fluctuated with intermittent peaks throughout the study period. The percentage of female-to-female dispute homicides remained very small during the 25-year period with no cases in the final two periods, and for this reason, no significance test was run.

Figure 4b (and its corresponding Table 6 in the appendix) shows the relationship between gender and age with robbery homicides. Similar to dispute homicides, most robbery homicides constituted male-to-male homicides (on average, 70% of all robbery homicides). The proportion of male-to-male robbery homicides increased during the 1990s to 78% and remained stable during the 2000s before it declined to 53.6% in the final years of the analysis. This decrease is, however, statistically non-significant (*p* = 0.43). This is mostly likely due to the small numbers per period. The median age of male perpetrators throughout the 25-year study period hovered around the late 20 s (and confirmed by a not statistically significant relationship between age and the years of observation). The median age of the male victims increased throughout the study period, from early 40 s to 50 s in the final periods, and this increase is statistically significant (*r*_s_ = 0.52, *p* = 0.01).

In the beginning of the observation period, one out of four robbery homicides was committed by male perpetrators against female victims. This percentage increased to more than one-third towards the end of the study period, but this increase is also not statistically significant (*p* = 0.30). Amongst robbery homicides, the other two gender constellations (female-to-male and female-to-female) were relatively uncommon and remained stable throughout the study period.

Unlike dispute and robbery homicides, intimate partner homicides were mainly committed by male perpetrators killing their female (ex-)partners (on average, 81%) (see Fig. 4c and Table 7 in the appendix). The proportion of male-to-female intimate partner homicides remained stable throughout the 25-year study period (*p* = 0.76). The median age of male perpetrators hovered around the mid-30 s in the 1990s and statistically significantly increased up to early mid-40 s in the remaining years (*r*_s_ = 0.66, *p* = 0.00). Female victims were younger than their male perpetrators: In the 1990s, their median age was early 30 s but statistically significantly increased to early 40 s in the final three periods (*r*_s_ = 0.79, *p* = 0.00).

The second largest group included female-to-male intimate partner homicide (on average, 14%). This group decreased over the years, starting at 19% of all intimate partner homicides in 1992–1994 and steadily declined to 12.9% in 2013–2016, but this decrease was not statistically significant (*p* = 0.48). Male-to-male lethal violence constituted 4% of all intimate partner homicides. Over the years, an increase in male-to-male intimate partner homicide can be noticed, from 1.7% in early study periods to a peak of 8.5% in 2004–2006, before it declined in the final periods of study.

Figure 4d (Tables 7 and 8 in the Appendix) shows the relationship between gender and age with criminal milieu homicides. Nearly all criminal milieu homicides took place between males (on average, 95% in the 25-year period). Over time, the proportion of male-to-male homicides remained stable. The trendline of age of male perpetrators also shows stability with one peak in the period 2007–2009. With regard to male victims, we see a small increase in age: at the start of the observation period, male perpetrators were in their early 30 s, and by the end of the 25-year observation period, the median age of the male victims increased to mid-30 s. This increase is, however, not statistically significant (*p* = 0.71). Approximately 5% of all criminal milieu homicides took place between a male perpetrator and a female victim, and while it fluctuated throughout the years, no statistically significant increase or decrease was found (*p* = 0.99). The percentage female-to-male and female-to-female criminal milieu homicides remained very small during the 25-year period, and for this reason, no significance test was run.

Figure 4e (and its corresponding Table 9 in the appendix) shows the relationship between gender and age with other familial homicides. These types of homicide were mainly committed by males killing male family members (on average, 45.3%) or males killing female family members (on average, 32.8%). Females killing male and female family members occurred approximately 1 in 10 cases throughout the 25 years. While all four types of gender constellation fluctuate throughout the observation period, there is no statistically significant increase or decrease found. With regard to the median age, only the age of the male perpetrator was calculated, because the age of the victim had a very large age range due to how this group was operationalized. This group consisted of, amongst others, neonaticides, infanticides and parricides, and for this reason, calculating the median age would not reflect the diverse age range of this group. The median age of the male perpetrators shows a small increase from the start of the observation period to the final period. However, this increase is not statistically significant (*p* = 0.11).

Figure 4f (and Table 10 in the appendix) shows the relationship between gender and age with other homicides. These types of homicide were mainly committed by males killing males (on average, 50.4%) or males killing females (on average, 43%). Females killing males and females formed a very small group. While all male-to-male homicide shows an increase throughout the observation period and male-to-female homicides showed a decrease, both observed trends were not statistically significant (*p* = 0.12 and *p* = 0.18, respectively). Only the age of the male perpetrator was calculated, for the same reason as given for other familial homicides. Furthermore, the number of victims in each year was too small to draw meaningful conclusions from. The median age of these male perpetrators shows a small decrease from the start of the observation period (33 years in the period 1992–1994) to the final period (29 years in the period 2013–2016). However, this decrease is not statistically significant (*p* = 0.88).

## Discussion

The aim of this study was twofold: first, to conduct an in-depth assessment of the nationwide homicide trend in the Netherlands, in which we sought to establish to what extent trends in specific types of homicide could provide an explanation for the overall homicide trend. In doing so, we were the first to examine a long-term (1992–2016), detailed and disaggregated national homicide trend. In line with previous studies that assessed the Dutch homicide trend in shorter time spans (Ganpat & Liem, 2012; Liem et al., 2012), we too found an overall declining homicide rate. This decline runs parallel to other European countries, including Finland and Sweden (Lehti, 2014; Trägardh et al., 2016; UNODC, 2019).

However, Verkko’s laws, formulated almost 70 years ago, do not seem to provide a complete understanding of today’s variation in homicide rates. To reiterate, Verkko (1967) argued that a decrease in the overall homicide rate was related to a decline in male-to-male violence. Results showed that as homicide rates declined, we witnessed a percental decrease in the *typical* male-to-male homicides, such as dispute homicides and robbery homicides. However, this decrease was not statistically significant. For criminal milieu homicides, other familial homicides and other homicides, the male-to-male homicides remained relatively stable throughout the study period. This means that we need to look beyond gender to understand the homicide rates per subtype.

We did find a statistically significant decrease in median age of male perpetrators of dispute homicides and an increase in median age amongst male victims of robbery homicides. We also found an increase in median age amongst male perpetrators and female victims of intimate partner homicide.

However, we refrain from accepting or declining the possible significant role demographics play in the Dutch homicide trends for two reasons. First, only simple correlations were done, as the aim of this paper was to explore homicide on a disaggregated level and examine how demographics (separately) are related to the observed trends. Second, the overall perpetration and victimization rate amongst 20- to 39-year olds has decreased considerably in the past 25 years—a noteworthy finding considering the small percental increase of this age group in the general Dutch population—suggesting we need to explore this age group more thoroughly. We, therefore, argue for a more in-depth study where gender and age are combined and further explored per subtype.

We did find that certain typical *male* homicide subtypes—specifically, dispute and robbery homicides—were significantly related to the overall homicide drop. A possible explanation can be found in the lifestyle theory. Aebi and Linde (2014) argued that a societal change in lifestyle constituted an important driver for the decline, for a large part influenced by changes in the pervasive use of the Internet. According to the lifestyle theory, the likelihood that an individual will be victimized depends on his or her lifestyle. The lifestyle theory is developed upon several premises, one of which is the uneven distribution of criminal victimization across time and space, where crime occurs in high-risk places (e.g. public domains, nightlife venues) and at high-risk times (e.g. at night, drinking times) (see also Walters, 2014). This explanation gives bearing to our own disaggregated findings: since the early years of the new millennium, at-risk young males increasingly spend their leisure time indoors, using personal computers connected to the Internet. Spending more leisure time indoors goes hand in hand with spending less time in *risky* public spaces. It may be argued that these developments, taken together, have contributed to an overall reduction of the number of dispute homicides. The pervasive role of the use and accessibility of the Internet could also provide an explanation for the decline in robbery homicides, where we raise the question whether robbery homicides, where physical money and goods are stolen, are making way for robberies including digital currency at its target (Conteh & Royer, 2016).

We also observed a decline in intimate partner homicide. The vast majority of these homicides involved male-to-female violence. We should note that the drop in intimate partner homicide is not in line with what we would expect based on lifestyle theory alone. As the lifestyle theory proposes, spending more time indoors allows potential victims and perpetrators to stay away from high-risk places, but at the same time, it results in family members spending more time together indoors (Carbone-López, 2006). The unique characteristics of a family heighten the risk of violence and homicide (Gelles & Straus, 1979; Liem & Koenraadt, 2018). In such cases, the home may turn into a high-risk place as motivated perpetrators, potential victims and the absence of a capable guardian are present (Cohen & Felson, 1979). A good example is the current COVID pandemic, where families are forced to spend more time indoors due to stay-at-home measures. Exploratory, mainly Western, research shows signs of an uptick in family violence during this pandemic (e.g. Agüero, 2020; Bullinger et al., 2020; Piquero et al., 2020). Similarly, it may be argued that the likelihood for violence to persist is higher, thereby increasing the likelihood for lethal violence, as family members are unable to leave the home environment (Campbell et al., 2007; Hendricks, 2006). Future work on the effects of COVID-19 on domestic homicide, in particular, should assess this hypothesis.

Yet, while the lifestyle theory could be applicable for (an increase in) familial homicides in extreme situations, we found little evidence of its application on our Dutch homicide data, since we found a drop in intimate partner homicides. We argue that this theory has its limitations, because it focuses on the home situation as a high-risk place while other factors can act as a counterbalance. Possible factors are the increased professional help in the field of domestic violence, which may be one of the precursors of intimate partner homicide (Spencer & Stith, 2018), and increases in women’s willingness to report domestic abuse (Liem et al., 2018a). Furthermore, an explanation can be found in the overall lower marriage rates in recent years, or in the possibility of divorce, where the cycle of violence between partners is broken (the so-called ‘exposure reduction hypothesis’, see Dugan et al., 2003) and women’s improved economic and social status in Western society, making them less dependent on their (former) male partners. While prior research has found evidence of a backlash perspective (e.g. Whaley & Messner, 2002)—where intimate partner violence rises as males perceive loss of power or control due to an improved status of their female partners—our findings show the opposite. According to Liem and colleagues (Liem and et al., 2018a), who studied intimate partner homicide in the Netherlands in the period 2009–2014, it seems that the availability of divorce in the Netherlands might have had a positive influence on the drop in the number of intimate partner homicides. However, since the drop in intimate partner homicides is more substantial than the divorce rate, a complete answer needs to be sought in combination with aforementioned factors (Liem et al., 2018a).

A few subtypes of homicides remained relatively stable over time or revealed countertrends. The first one concerns other familial homicides (not including intimate partner homicides) that were (only just) not significantly related to the overall homicide trend. A possible explanation for this lack of relationship with the overall homicide trend is that this subtype comprises of several types of familial homicide—with the exception of intimate partner homicide—such as filicide (the killing of a child), parricide (the killing of a parent), siblicide (the killing of a sibling) and familicide (the killing of multiple family members). Each of these subtypes tends to have different underlying characteristics and precursors (for an overview, see Liem & Koenraadt, 2018) than homicides taking place outside the family, and each might affect the overall homicide rates differently. However, it is difficult to disaggregate this subtype into more subtypes, as samples will become too small to base any conclusions on. The same applies for the subtype ‘other homicides’; this subtype constitutes the smallest group, accounting for 5.8% of the total sample in the 25-year observation period. The small *N* disallowed us to draw any meaningful conclusions from this subtype of homicide.

In this study, we also found that trends in criminal milieu homicides were not significantly related to the general homicide trend but, instead, slightly increased over time. This can be attributed to their close relation to the presence and operations of drug markets in recent years. Even though the relationship between drugs and homicide has received little academic attention in Europe (De Bont et al., 2018), here, we can reflect on possible reasons why such homicides have not declined as other types of homicide have throughout the study period. A plausible answer seems to lie in the nature of drug markets in the Netherlands, which is known as a country of production when it comes to both cannabis and synthetic drugs; at the same time, it is characterized as a transit country for cocaine and heroin (EMCDDA, 2019). According to latest reports (EMCDDA, 2019), there has been an increase in the number of dismantled production labs and drug seizures over the past few years, which implies that drug markets have been expanding in the Netherlands. Furthermore, online drug trafficking has been on the rise in recent years (EMCDDA, 2019). Taken together, these factors are thought to play a key role in escalating levels of drug-related violence in the Netherlands: Specifically, in 2017, there was a total of 31 contract killings as a result of conflicts related to drug trafficking, and this number has been stable since 2000 (Van Laar & Van Gestel, 2018). The active drug market—in terms of both production and trafficking—and systemic violence associated with such markets could be relatively immune to factors responsible for declines in other types of violence.

### Limitations

Despite its longitudinal and detailed nature, using the Dutch Homicide Monitor is not without problems. First, case-based information is only available since 1992, while adequate digitalization and coding of homicide cases only started at the beginning of the twenty-first century. This may have resulted in a relatively higher prevalence of missing data for homicide cases in these early periods. Second, caution must be exercised when disaggregating homicide into subtypes. There are many different classification systems (see also Skott, 2019). Although our classification stems from a validated coding system and has also been used in several (inter)national studies (e.g. Liem et al., 2013, 2018), due to the applied hierarchy (the relationship between perpetrator and victim determines first the type of homicide, followed by the motive), the number of cases in certain subtypes of homicide has remained very small. These include, for example, homicides that were driven by a profound influence of mental illness. Zooming in on these cases revealed that these mostly involved family members of the victims and, as such, were categorized as intimate partner or other family homicides, and not as homicide characterized by a profound influence of mental illness. The same applies for sexual homicides. In future research, when the focus is on specific subtypes, such as sexual homicides or homicides driven by a profound influence of mental illness, the motive should precede the relationship between perpetrator and victim. Lastly, our data on homicide types may not provide a complete picture of the homicide drop on a disaggregated level, since almost one out of five cases has remained uncleared (see also Liem et al., 2018a). Hence, an adequate classification based on victim-perpetrator relationship and motive for this group cannot be made. Furthermore, it is also important to note that the “missingness” of victim-perpetrator relationship in uncleared cases does not appear to be random (Rosenfeld & Fox, 2019). In our study, criminal milieu homicides oftentimes remained uncleared, more so than other subtypes of homicide. In future analyses, imputation techniques should be considered, for example by using data on hard-to-solve cases that turned out to be cleared. Information from such cases could then be used for imputing data on missing cases (Roberts et al., 2018).

## Conclusion

To conclude, this is the first study to assess nationwide, disaggregated homicide trends over a 25-year period. Using a unique, detailed dataset, spanning a quarter of a century of homicide data allowed us to assess type-specific homicide trends and the relationship between gender and age within these trends. In doing so, we have not only been able to examine the overall declining trend in homicide but were also able to more closely assess the types of homicide associated with the decline. Our findings highlight the explanatory potential of disaggregated homicide data to allow us to further unravel the downward trend. And while gender and age offered starting points in understanding the disaggregated trends, other explanations that are beyond the scope of this article need to be taken into consideration in future work in this area. We find promise in Eisner’s (2008) suggestion: “my favourite candidate for explaining the downturn would be […] culture, the only phenomenon that travels fast enough to affect such vast areas roughly simultaneously” (p. 311). Eisner (2008) further argued that we need to examine the change in culturally embedded images of conducting life, such as how to raise children and the re-emphasis of values such as self-control and respect. These may indeed prove to be important contributing factors to our observed decline in intimate partner homicides. For dispute and robbery homicides, future studies could examine exactly how the Internet has adapted the lifestyle amongst young men through more in-depth qualitative research. Such follow-up studies would aid us in unravelling the homicide drop further and could provide us with tools in the form of possible subtype-based prevention strategies to maintain the decline in the homicide rate.

## Data Availability

Due to the sensitivity of the data, only aggregated data can be made available.

## Appendix

**Table 1:** Overall homicide rate per 100,000 inhabitants and percentage gender constellation in the period 1992–2016

	M perp − M vic (%)	M perp − F vic (%)	F perp − M vic (%)	F perp − F vic (%)
1992–1994	57.6	29.5	5.8	2.0
1995–1997	55.9	32.0	4.7	5.2
1998–2000	63.1	27.5	5.2	2.6
2001–2003	56.7	31.7	6.2	3.1
2004–2006	59.6	30.0	6.3	2.4
2007–2009	57.9	29.2	7.1	3.3
2010–2012	55.4	34.0	6.8	2.9
2013–2016	56.6	34.0	6.2	1.8

**Table 2:** Homicide rate of perpetrators per age group per 100,000 inhabitants in the period 1992–2016

	Younger than 20 years	20–39 years	40–64 years	65–79 years	80 years and older
1992–1994	0.74	3.18	0.85	0.09	0.07
1995–1997	0.72	3.54	0.99	0.08	0.07
1998–2000	0.74	3.98	0.98	0.06	0.07
2001–2003	0.81	4.00	1.13	0.10	0.19
2004–2006	0.70	3.32	0.95	0.08	0.12
2007–2009	0.46	2.73	0.89	0.09	0.00
2010–2012	0.43	2.44	0.88	0.17	0.05
2013–2016	0.12	1.20	0.39	0.02	0.10

**Table 3:** Homicide rate of victims per age group per 100,000 inhabitants in the period 1992–2016

	Younger than 20 years	20–39 years	40–64 years	65–79 years	80 years and older
1992–1994	0.63	2.93	1.43	0.72	0.95
1995–1997	0.78	2.84	1.54	0.85	1.38
1998–2000	0.76	2.55	1.35	0.55	0.47
2001–2003	0.63	2.51	1.44	0.46	0.75
2004–2006	0.73	1.85	1.13	0.56	0.81
2007–2009	0.62	1.68	1.06	0.35	0.49
2010–2012	0.60	1.56	1.03	0.50	0.80
2013–2016	0.24	0.76	0.43	0.34	0.21

**Table 4:** Disaggregation of homicide rate per 100,000 inhabitants by subtype, in the period 1992–2016

	IPH	Other familial killings	Criminal milieu	Robbery	Disputes	Other
1992–1994	0.26	0.13	0.17	0.15	0.46	0.09
1995–1997	0.27	0.17	0.14	0.13	0.44	0.11
1998–2000	0.23	0.17	0.19	0.11	0.38	0.11
2001–2003	0.28	0.16	0.19	0.11	0.30	0.07
2004–2006	0.19	0.21	0.16	0.10	0.26	0.06
2007–2009	0.18	0.15	0.12	0.08	0.28	0.04
2010–2012	0.23	0.14	0.16	0.08	0.21	0.06
2013–2016	0.15	0.11	0.19	0.05	0.16	0.06

**Table 5:** Median age and gender constellation of dispute homicides in the period 1992–2016

	Age M perp (median)	Age M vic (median)	M perp − M vic (%)	M perp − F vic (%)	F perp − M vic (%)	F perp − F vic (%)
1992–1994	28.5	31	93.7	5.8	0.0	0.5
1995–1997	29	30	92.0	5.9	1.1	1.1
1998–2000	31	31.5	89.0	7.3	1.8	1.8
2001–2003	30	33	86.4	8.3	3.8	1.5
2004–2006	29	32	90.9	7.4	0.8	0.8
2007–2009	30	35	80.9	12.8	3.5	2.8
2010–2012	31.5	33.5	94.1	5.0	1.0	0.0
2013–2016	33	35	87.1	9.9	3.0	0.0

**Table 6:** Median age and gender constellation of robbery homicides in the period 1992–2016

	Age M perp (median)	Age M vic (median)	M perp − M vic (%)	M perp − F vic (%)	F perp − M vic (%)	F perp − F vic (%)
1992–1994	27	42	67.4	27.9	4.7	0.0
1995–1997	25	47	68.0	24.0	4.0	4.0
1998–2000	29.5	39	77.8	16.7	5.6	0.0
2001–2003	27.5	42.5	78.3	15.2	6.4	0.0
2004–2006	26	52.5	75.6	19.5	4.9	0.0
2007–2009	24	49	77.1	20.0	2.9	0.0
2010–2012	29	58	65.9	29.3	4.9	0.0
2013–2016	29	51.5	53.6	35.7	3.6	7.1

**Table 7:** Median age and gender constellation of intimate partner homicides in the period 1992–2016

	Age M perp (median)	Age F vic (median)	M perp − M vic (%)	M perp − F vic (%)	F perp − M vic (%)	F perp − F vic (%)
1992–1994	36	30	1.7	79.3	19.0	0.0
1995–1997	35	32	4.5	87.1	8.3	0.0
1998–2000	36	33.5	1.9	79.6	18.4	0.0
2001–2003	36.5	35	5.1	81.2	11.6	2.2
2004–2006	39	35	8.4	78.9	12.6	0.0
2007–2009	45	42	2.3	81.4	16.3	0.0
2010–2012	45	37	6.0	79.5	14.5	0.0
2013–2016	42.5	41	3.9	82.4	13.7	0.0

**Table 8:** Median age and gender constellation of criminal milieu homicides in the period 1992–2016

	Age M perp (median)	Age M vic (median)	M perp − M vic (%)	M perp − F vic (%)	F perp − M vic (%)	F perp − F vic (%)
1992–1994	31.5	32	95.9	4.1	0.0	0.0
1995–1997	28	32	93.2	2.3	2.3	2.3
1998–2000	30	31	98.5	1.5	0.0	0.0
2001–2003	31.5	33	91.4	7.1	1.4	0.0
2004–2006	31	33	91.4	8.6	0.0	0.0
2007–2009	37	36	88.9	11.1	0.0	0.0
2010–2012	31	30.5	97.4	2.6	0.0	0.0
2013–2016	32.5	35	95.7	4.3	0.0	0.0

**Table 9:** Median age and gender constellation of other familial homicides in the period 1992–2016

	Age M perp (median)	M perp − M vic (%)	M perp − F vic (%)	F perp − M vic (%)	F perp − F vic (%)
1992–1994	30.5	45.2	32.3	8.1	14.5
1995–1997	29.5	43.8	39.3	7.9	9
1998–2000	30	50	29.2	9.7	11.1
2001–2003	26	37.5	37.5	11.4	13.6
2004–2006	32	44.1	34.3	11.8	9.8
2007–2009	29	46.5	26.8	11.3	15.5
2010–2012	32	45.2	30.1	12.3	12.3
2013–2016	34	51.9	29.9	10.4	7.8

**Table 10:** Median age and gender constellation of other homicides in the period 1992–2016

	Age M perp (median)	M perp − M vic (%)	M perp − F vic (%)	F perp − M vic (%)	F perp − F vic (%)
1992–1994	33	44.1	50	2.9	2.9
1995–1997	28.5	46.5	48.8	2.3	2.3
1998–2000	27	44.2	46.2	3.8	5.8
2001–2003	30	50	42.9	3.6	3.6
2004–2006	28	56.7	33.3	6.7	3.3
2007–2009	30	57.9	36.8	5.3	0
2010–2012	26	53.6	42.9	0	3.6
2013–2016	29	58.3	36.1	2.8	2.8

## Footnotes

1 The EHM category ‘other in non-criminal milieu’ (value 11, see also the EHM coding manual in Granath et al., 2011) is included in this subtype. We have, however, termed this subtype ‘non-criminal disputes’ as in all cases, the victim and perpetrator had a dispute that resulted in the homicide. For this reason, nightlife violence was also included in this subtype, as in these cases, the homicide was always the result of a dispute.

2 Statistics Netherlands only has information on the population size per year for these five age groups.
